# Molecular modeling and simulation approaches to characterize potential molecular targets for burdock inulin to instigate protection against autoimmune diseases

**DOI:** 10.1038/s41598-024-61387-7

**Published:** 2024-05-17

**Authors:** Huma Farooque Hashmi, Xu Xuan, Kaoshan Chen, Pengying Zhang, Muhammad Shahab, Guojun Zheng, Youssouf Ali Younous, Ahmad Mohammad Salamatullah, Mohammed Bourhia

**Affiliations:** 1 School of Life Science and National Glycoengineering Research Center, Shandong University, Qingdao, 266237 China; 2 State Key Laboratories of Chemical Resources Engineering, Beijing University of Chemical Technology, Beijing, 100029 People’s Republic of China; 3 Evangelical College, BP 1200, N’Djamena, Chad; 4 Department of Food Science and Nutrition, College of Food and Agricultural Sciences, King Saud University, P.O. Box 2460, 11451 Riyadh, Saudi Arabia; 5 Laboratory of Biotechnology and Natural Resources Valorization, Faculty of Sciences, Ibn Zohr University, 80060 Agadir, Morocco

**Keywords:** Inulin, Structural modeling, Simulation, Docking, Free energy, Computational biology and bioinformatics, Drug discovery

## Abstract

In the current study, we utilized molecular modeling and simulation approaches to define putative potential molecular targets for Burdock Inulin, including inflammatory proteins such as iNOS, COX-2, TNF-alpha, IL-6, and IL-1β. Molecular docking results revealed potential interactions and good binding affinity for these targets; however, IL-1β, COX-2, and iNOS were identified as the best targets for Inulin. Molecular simulation-based stability assessment demonstrated that inulin could primarily target iNOS and may also supplementarily target COX-2 and IL-1β during DSS-induced colitis to reduce the role of these inflammatory mechanisms. Furthermore, residual flexibility, hydrogen bonding, and structural packing were reported with uniform trajectories, showing no significant perturbation throughout the simulation. The protein motions within the simulation trajectories were clustered using principal component analysis (PCA). The IL-1β–Inulin complex, approximately 70% of the total motion was attributed to the first three eigenvectors, while the remaining motion was contributed by the remaining eigenvectors. In contrast, for the COX2–Inulin complex, 75% of the total motion was attributed to the eigenvectors. Furthermore, in the iNOS–Inulin complex, the first three eigenvectors contributed to 60% of the total motion. Furthermore, the iNOS–Inulin complex contributed 60% to the total motion through the first three eigenvectors. To explore thermodynamically favorable changes upon mutation, motion mode analysis was carried out. The Free Energy Landscape (FEL) results demonstrated that the IL-1β–Inulin achieved a single conformation with the lowest energy, while COX2–Inulin and iNOS–Inulin exhibited two lowest-energy conformations each. IL-1β–Inulin and COX2–Inulin displayed total binding free energies of − 27.76 kcal/mol and − 37.78 kcal/mol, respectively, while iNOS–Inulin demonstrated the best binding free energy results at − 45.89 kcal/mol. This indicates a stronger pharmacological potential of iNOS than the other two complexes. Thus, further experiments are needed to use inulin to target iNOS and reduce DSS-induced colitis and other autoimmune diseases.

## Introduction

The immune system of our body provides protection to all body organs and maintains the balanced homeostatic environment inside the body required for normal biochemical functioning. The immune systems of different organs are also well-organized and capable of producing a large number of surveillance cells and biochemical immune molecules that can effectively halt pathogens causing infection in any particular area^1^. In normal, healthy physiological states, the immune system coordinates with the metabolic systems of the body and responds to any internal or external changes, ensuring the organism's need for adaptation^2^. Cytokines are key components of the immune system, playing a crucial role in the regulation of immune responses and inflammation. They are produced in response to diseases and act by interacting with either cytokine inhibitors or soluble cytokine receptors. Cytokines comprise multiple proteins, including interleukins, monokines, lymphokines, and chemokines^3,4^. Cytokines and their clinical importance are explored based on their pro- and anti-inflammatory activities^4^.

Cytokines can either be proinflammatory, playing a role in provoking diseases when their expression leads to inflammatory conditions. For example, tumor necrosis factor α (TNF-α) is implicated in diseases such as rheumatoid arthritis and Crohn’s disease. On the other hand, some cytokines have anti-inflammatory properties, working to minimize inflammation and promote healing in pathological conditions. These cytokines can stimulate the immune system and possess the ability to combat foreign pathogens^5,6^. The pro-inflammatory cytokines, such as interleukin (IL)-1, TNF-alpha, and IL-6, are typically released in response to disease conditions. They initiate a diverse range of cell signaling events that can lead to necrosis or apoptosis^7,8^. Some cytokine receptors, such as those for IL-1, tumor necrosis factor-alpha, and IL-18, act as inhibitors of proinflammatory cytokines^9^. The IL-1 receptor antagonist, IL-4, IL-6, IL-10, IL-11, and IL-13 constitute crucial anti-inflammatory cytokines. Additionally, some cytokines, for example, TNF-alpha, have both anti-inflammatory and proinflammatory effects. TNF-alpha plays a major role in homeostasis and the maintenance of the immune system, but during the middle and old age stages of life, it can contribute to chronic inflammation, being abundantly expressed in conditions such as rheumatoid arthritis and bipolar disorder^10–12^.

The inducible nitric oxide synthase (iNOS) is another inflammatory factor that is highly upregulated in cancer and is involved in photodynamic therapy for cancer. It directly produces nitric oxide (NO)^13,14^. Besides, the cyclooxygenase (COX) and its downstream effector molecules also has great significance. COX-2 is increased in several of brain cells following seizure induction, leading to a high release of proinflammatory mediators, specifically prostaglandins (PGs). This increase in COX-2 and subsequent release of PGs can aggravate seizure severity and contribute to proteinoid formation during mega-karyopoiesis^15,16^. While the interleukin-1β (IL-1β) cytokine is essential for the host response and is known to confer resistance to pathogens, it can also intensify damage in chronic diseases or acute injuries^17^. In fact, the balance between cytokines is important for normal body function, and they also serve as good biomarkers to study disease status. Thus, the role of cytokines has been notably crucial, and more focus has been placed on inhibiting cytokines that can be damaging to the host, particularly during severe infections. In this context, medicinal plants are an important source of anti-inflammatory compounds and are generally found to be an effective alternative to synthetic medications, which often come with numerous harmful effects^18,19^.

Burdock (*Arctium lappa *L.) is a traditional Chinese medicinal plant for many years which is also cultivated in a diverse range of climates including the China, Japan, Korea, Europe, and United Sates^20^. It is a famous vegetable in many countries and their roots are used as source of food. Burdock has potent active ingredients like tannin, beta-eudesmol, inulin, lappaol, etc. Which have many antioxidants, antiallergic, antidiabetic, immunoregulatory, and anti-inflammatory activities^21–23^. The inulin component is a member of the fructans family and is essentially a water-soluble storage polysaccharide used as a prebiotic, fat and sugar replacement, with positive effects on intestinal health. It is also capable of enhancing immunity both in vitro and in vivo with no negative health effects. Chitosan (CHI)-based inulin nanoparticles also exhibit strong antioxidant and antimicrobial activities^24^. For instance, we previously reported that the inulin from Burdock root (BFO) can be used to downregulate mRNAs that control the expression of several inflammatory proteins such as iNOS, COX-2, TNF-α, IL6, and IL-1β^25^. Although these targets were revealed, further validations, either experimental or computational, are necessary to confirm the activity of inulin against these specific targets. Therefore, in this study, we used state-of-the-art computational methods to characterize a specific target for inulin in DSS-induced colitis conditions. Through structural modeling and molecular simulation approaches, the interaction paradigm of inulin was explored with iNOS, COX-2, TNF-alpha, IL6, and IL-1β. Based on the docking scores and interaction with key essential amino acids, the complexes with the best docking scores were subjected to all-atoms simulation to investigate dynamic stability, structural packing, flexibility, and other features in a highly dynamic environment. We further validated the best scoring complexes by calculating the binding free energy for the top three hits and reported hydrogen bonding variations as well. Our results potentially identify iNOS as the best potential target for inulin, and therefore further investigations are demanded to validate the activity of inulin for prospective clinical usage.

## Materials and methods

### Preparation of ligands and receptors

Initially, we downloaded the crystal structures of iNOS (PDB ID; 3HR4), COX-2 (PDB ID; 5IKQ), TNF-alpha (PDB ID; 2AZ5), IL6 (PDB ID; 7NXZ), and IL-1β (PDB ID; 4G6J) from the RCS PDB database. Subsequently, these structures were processed for further analysis by removing any extra attached water molecules and were saved in .pdbqt format^26^. The ligand structures were obtained from the PubChem database, and their energy was minimized. Subsequently, the resulting structure was subjected to an energy minimization algorithm using the Swiss-PdbViewer v.4.10 software program^27^. To ensure the protein was suitable for docking with AutoDock Vina, we first obtained its three-dimensional structure and meticulously refined it. This involved addressing any missing atoms or loops within the protein. We then optimized the protein structure by eliminating water molecules and introducing essential hydrogen atoms. The refined protein structure was saved in the PDBQT file format, which AutoDock Vina specifically requires for protein input. Similarly, we acquired the three-dimensional structure of the ligand and followed a comparable procedure for its preparation. The ligand underwent optimization by adding hydrogen atoms and eliminating unfavorable conformations. Additionally, partial charges were assigned to the ligand atoms, commonly employing force field-based methods. Finally, the prepared ligand structure was saved as a PDBQT file format, ready to be docked with the protein using AutoDock Vina.

### Molecular docking

For protein–ligand docking, various computational strategies are routinely employed, especially for discovering small drug-like molecules. In this study, we utilized AutoDock Vina to examine the binding interactions between Inulin and iNOS, COX-2, TNF-alpha, IL6, and IL-1β^28^. AutoDock Vina employs an exhaustiveness parameter, which determines the thoroughness of the search algorithm during the docking process. The amino acid residues that constitute the active site of the protein were firstly delineated, which a grid box of size x = 15.1651137855 Å, y = 19.301165182 Å, and z = 13.9173764782 Å, with a center dimension of x = 18.4802485932, y = -18.9999491012, and z = -50.2219055 were set to define the active site. Conversely, a blind docking protocol was employed to study the interactions, the grid box with size x = 62.2440970136 Å, y = 77.8606218435 Å, and z = 62.064913585 Å, with a center dimension of x = 31.0115192852, y = 71.8214808942, and z = 80.3147800551 was found to cover every residue of the protein. The docking procedure was then executed using an exhaustiveness of 8. This allows for a more exhaustive exploration of the binding landscape and increases the chances of identifying optimal binding modes. In our approach, we set the exhaustiveness parameter to 16 to enhance binding accuracy. For each case, 10 different poses were initially generated, and based on the best docking score, the best conformation was selected to examine the protein–ligand binding mechanism using PyMol^29–31^.

### Molecular dynamics simulation

To understand the structural inhibition effect of Inulin on iNOS, COX-2, and IL-1β cytokines, the docked complexes were inspected by a molecular dynamics’ simulation procedure using Amber 20 simulation software. We initially employed the “FF14SB” force field to define the protein^32^, and then added a cubic box of the TIP3P water model (with a buffer distance of 10 Å) to solvate the protein. Both negative and positive counter ions (Na^+^ and Cl^−^) were further added to balance the system^33^. To ensure the system is fit for optimal performance, a total of 1000 energy minimization steps were performed including the 500 steepest descent and 500 conjugate gradient steps. The system was steadily heated from 0 to 325 (K)^34^ and the hydrogen bonds are subsequently controlled via the SHAKE method. During the MD simulations procedure, the Langevin dynamics approach was used i.e., with pressure of 1 atm and temperature of 310 K^35^. At a constant pressure and temperature, each system was first equilibrated for 10 ns (ns) time. Following that, the MD simulation was conducted for 150 ns (ns) total time at a constant temperature of 325 K^36^. Finally, data visualization and analysis were performed with the help of Origin Software. We further thoroughly examined the conformational alterations that eventuated during simulations. To analyze these alterations, we utilized CPPTRAJ module within AMBER20 to investigate several factors namely root-mean-square deviation (RMSD), root-mean-square fluctuation (RMSF), Radius of gyration (RoG) and Principal component analysis (PCA). The Radius of gyration (RoG) is a valuable measure for analyzing protein size and compaction^37^.

### Binding free energy calculation

To evaluate the binding strength in molecular simulation, we employed the MM/PBSA.py script to calculate the binding free energy^38,39^. This method, known for its broad applicability, offered several advantages over other approaches, including computational efficiency and reduced time requirements. For each complex, the van der Waals (vdW), electrostatic, solvent-accessible surface area (SASA), and as well as the generalized Born (G.B.) components were determined on the basis of the entire simulation trajectory. This approach combines molecular mechanics simulations, which describe the interactions between atoms, with implicit solvent models, which describe the interactions between the protein and solvent^40–44^. Hence, we also applied this approach here to accurately compute the total binding free energy of the protein–ligand complexes. Mathematically the binding free energy can be estimated as:

1 $$\Delta G(bind) = G(complex) - [G(receptor) + G(ligand)]$$

Different contributing components of total binding energy were calculated by the following equation:

2 $$G=Gbond + Gele +GvdW +Gpol +Gnpol$$

It has a wide range of applications i.e., used to estimate the binding energy for proteins in different studies including SARS-CoV-2 and neurological disorders^43,45–49^.

### Principal component analysis (PCA)

Each clustering of the trajectory was created on the basis of calculation and diagonalization of the covariant atomic fluctuation using the principal component analysis (PCA)^50,51^. In PCA analysis both diagonal eigenvectors and analogous eigenvalues values were extracted. The principal components (PCs) that provide the information on atom motions and displacement are also known as eigenvectors and has already been discussed previously^52,53^. The following equation was used for the cluster trajectory analysis.

3 $$DG\left(X\right)1/4 kBT ln P\left(X\right)$$

X denotes the response of two principal components, kB is Boltzmann constant, while the P(X) represents the dispersion of the framework's likelihood. The Free energy landscape (FEL) was built from the two PCs.

## Results and discussion

**Figure 1:**
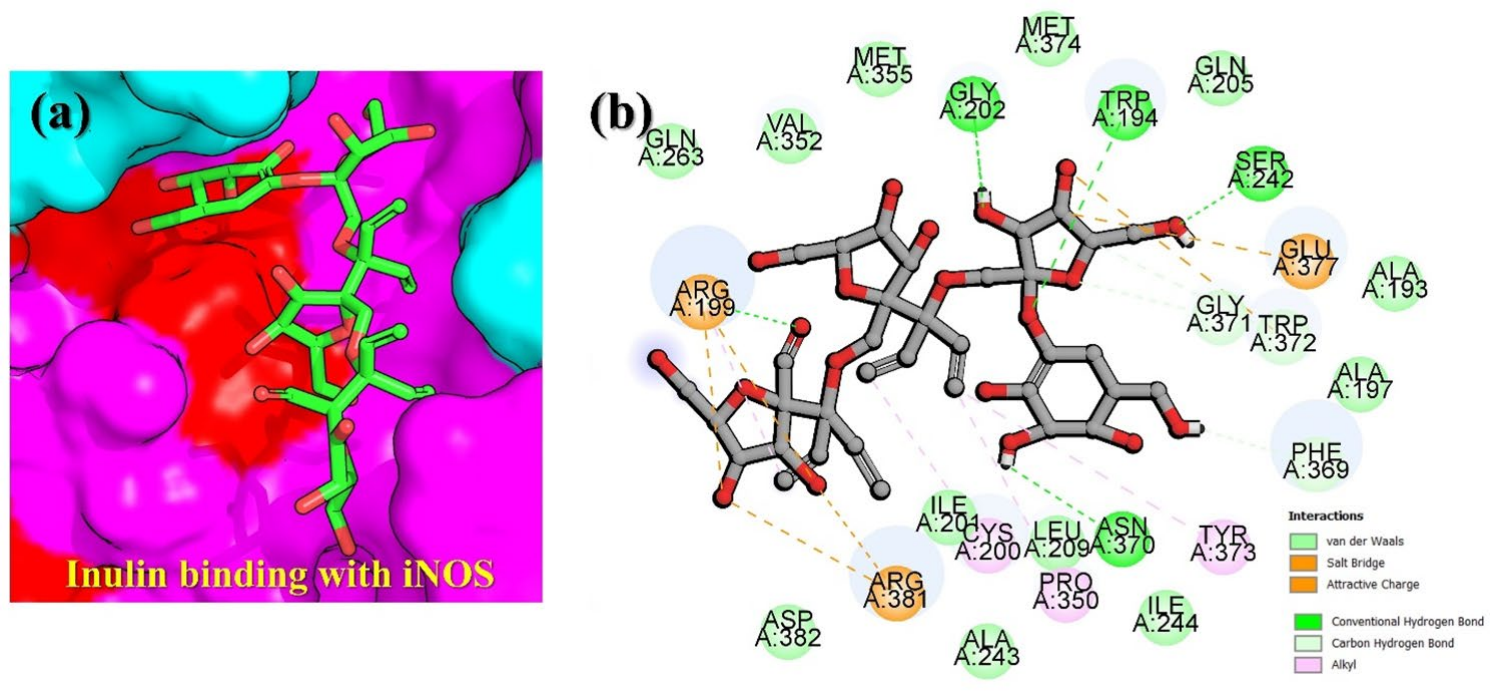
Interaction paradigm of inulin with iNOS. (**a**) 3D binding mode of inulin in the active site cavity of iNOS. (**b**) Show the 2D binding and different interactions in the iNOS–inulin complex.

**Figure 2:**
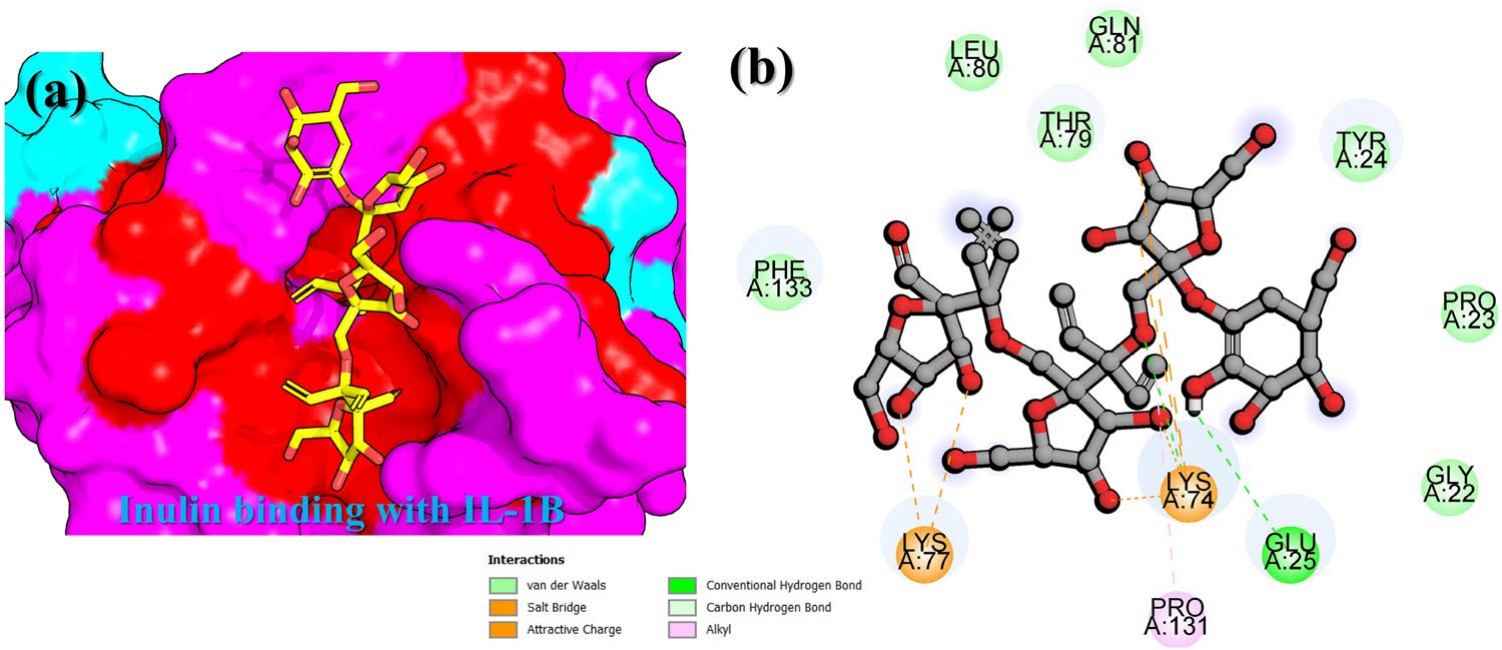
Interaction paradigm of inulin with IL-1B. (**a**) 3D binding mode of inulin in the active site cavity of IL-1B. (**b**) Show the 2D binding and different interactions in the IL-1B–inulin complex.

In the current study, molecular modeling and all-atoms simulation approaches have been integrated to computationally validate the potential target for Inulin, which is an active ingredient of Burdock. Reduced expression of IL-6, IL-1β, TNF-alpha, iNOS and COX-2 in the experimental analysis of burdock extract revealed these receptors as the potential targets in immunocompromised diseases and particularly DSS-induced colitis^54–57^. We employed structural and simulation methods to investigate the binding of Inulin with these targets and further validated by using binding free energy approach. The docking results demonstrated that the iNOS–Inulin exhibit the best docking energy of − 7.54 kcal/mol followed by IL-1β with the docking score of − 5.08 kcal/mol. Moreover, the docking results for COX-2 and TNF-alpha complexes revealed to be − 4.80 kcal/mol, respectively. For IL-6, the docking energy was calculated to be − 4.00 kcal/mol, which is the lowest among all. The biomolecular interaction paradigm of each complex was explored and the interaction features were comprehensively explored. For instance, the iNOS–Inulin complex reported various group of interactions including van der Waals, hydrogen bonds, hydrophobic and charge interactions. The iNOS–Inulin complex stands the best with 8 hydrogen bonds established with Trp194, Arg199, Ile201, Gly202, Ser242, Phe369, Asn370, Gly371 and Trp372, while the salt-bridges, pie-anion, alkyl, pie-alkyl and attractive charges contacts were also frequently observed. The 3D binding mode of inulin the binding cavity of iNOS is given in Fig. 1a, while the 2D detailed interaction pattern of inulin with iNOS is shown in Fig. 1b. The second-best docking complex was IL-1B–Inulin, which established two hydrogen bonds and multiple hydrophobic interactions. Among the hydrogen bonds Glu25 and Lys74 residues while the Lys74, Lys77 and Pro131 residues were involved in hydrophobic interactions. The residues Lys74 and Lys77 were particularly involved in salt bridges while Pro131 connected with inulin through an alkyl interaction. For instance, these residues are also reported to act as inhibitor hotspots for other drug targets^58^. The 3D binding mode of inulin the binding cavity of IL-1B is given in Fig. 2a, while the 2D detailed interaction pattern of inulin with IL-1B is shown in Fig. 2b.

**Figure 3:**
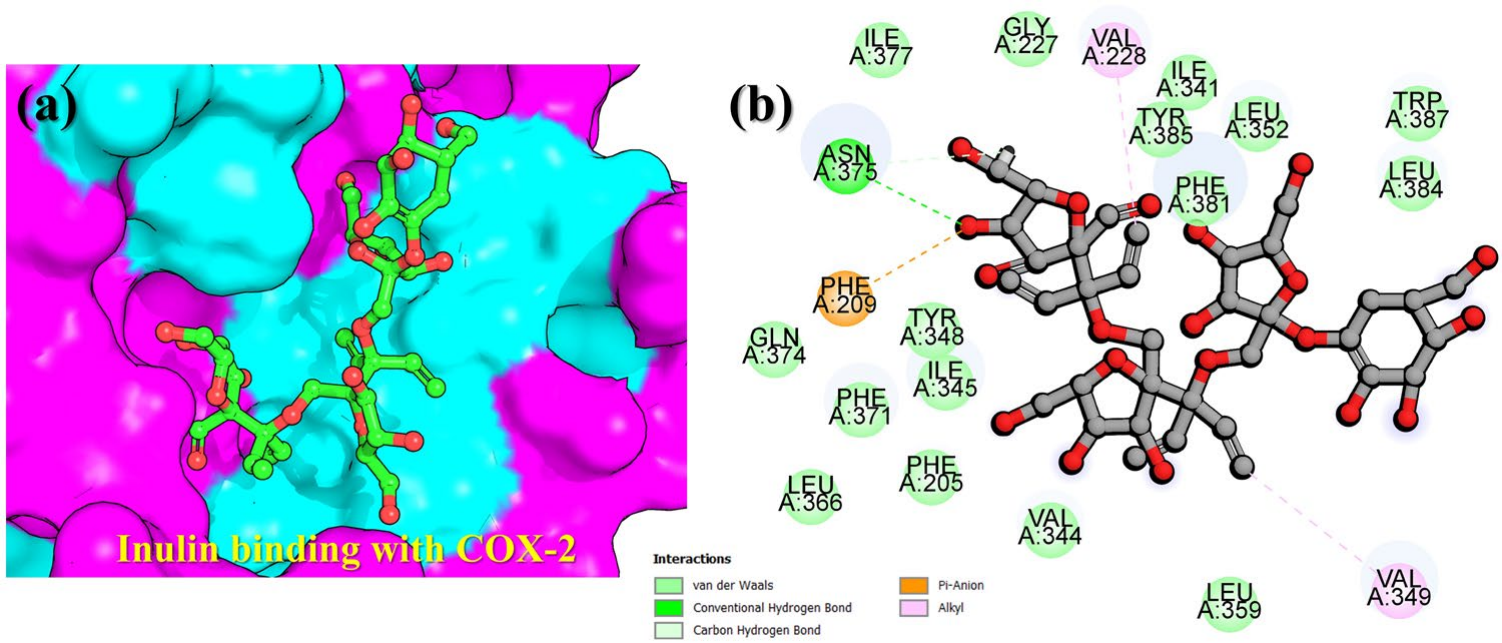
Interaction paradigm of inulin with COX-2. (**a**) 3D binding mode of inulin in the active site cavity of COX-2. (**b**) Show the 2D binding and different interactions in the COX-2–inulin complex.

Moreover, the COX2 and TNF-alpha demonstrated a similar docking score of − 4.80 kcal/mol. The interaction paradigm of COX2–Inulin revealed a single hydrogen bond with Asn375, while the pie-anion interaction was established by Phr209 and alkyl interaction was reported with Val228 and Val349. Although the complex demonstrated relatively good docking score but the interactions of the ligand with the active site residues are relatively lower in numbers. Interestingly our results strongly corroborate with previous literatures where blocking of these residues in the COX2 and TNF receptor led to the inhibition of the pathological role of these proteins^59–61^. The 3D binding mode of inulin the binding cavity of COX2 is given in Fig. 3a, while the 2D detailed interaction pattern of inulin with COX2 is shown in Fig. 3b.

**Figure 4:**
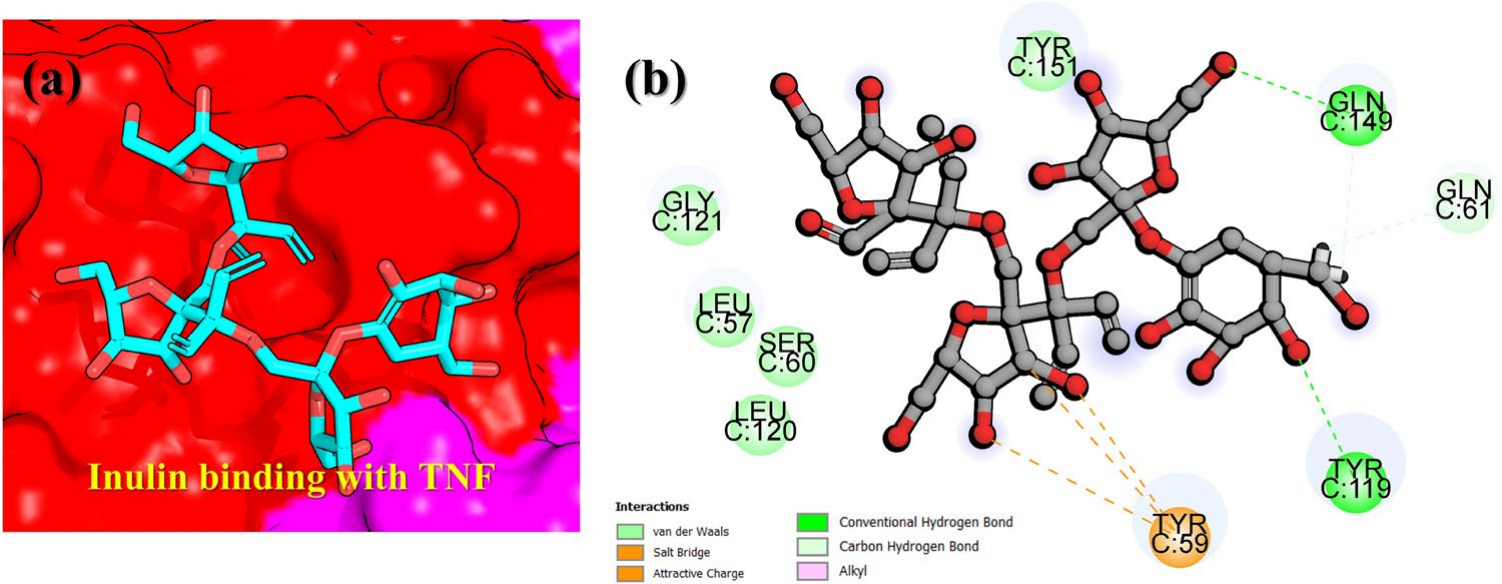
Interaction paradigm of inulin with TNF-alpha. (**a**) 3D binding mode of inulin in the active site cavity of TNF-alpha. (**b**) Show the 2D binding and different interactions in the TNF-alpha–inulin complex.

On the other hand, the TNF-alpha bound with inulin reported two stronger hydrogen bonds with Tyr119 and Gln149, while three salt bridges by Tyr59 were observed. Although the number of bonds is more in number than the previous compounds but still the binding energy align with each other. The 3D binding mode of inulin the binding cavity of TNF-alpha is given in Fig. 4a, while the 2D detailed interaction pattern of inulin with TNF-alpha is shown in Fig. 4b.

**Figure 5:**
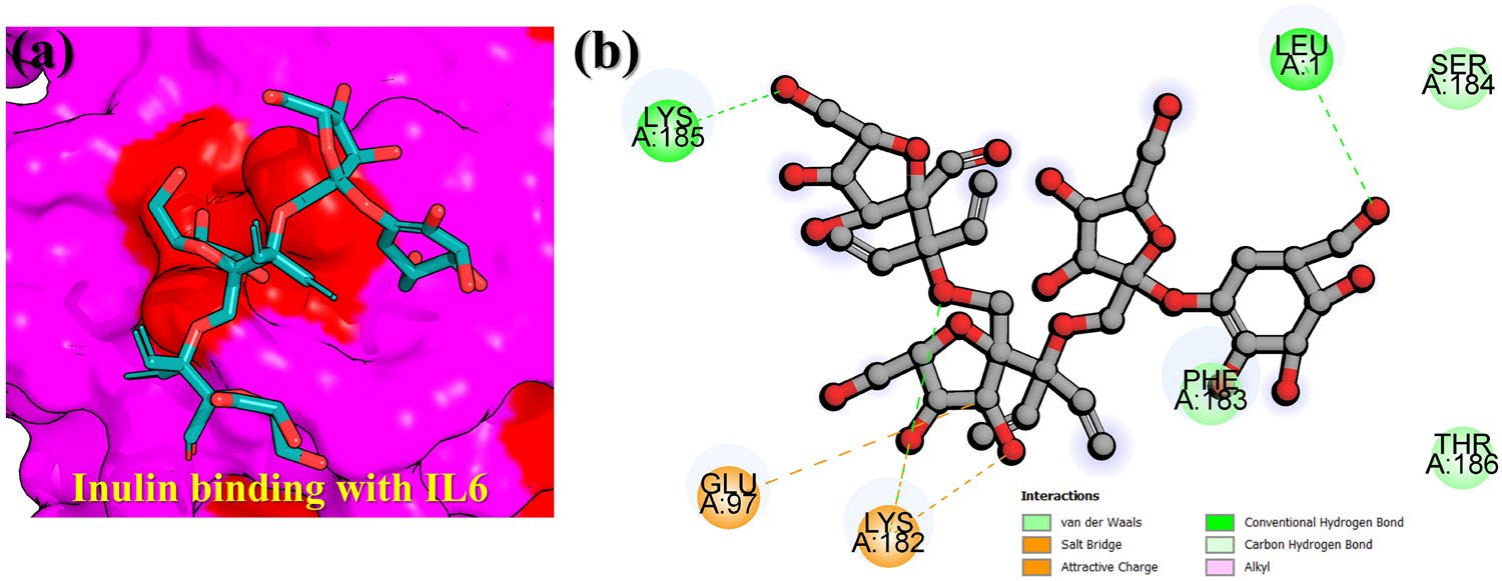
Interaction paradigm of inulin with IL6. (**a**) 3D binding mode of inulin in the active site cavity of IL6. (**b**) Show the 2D binding and different interactions in the IL6–inulin complex.

To see how the binding of inulin occur with IL-6 the docking was also performed against the active site residues of IL-6. The docking results revealed that Leu1 and Lys185 established robust hydrogen bonds with inulin, while the salt bridges were reported with Glu97 and Lys182 residues. Lys182 was also observed to have a hydrogen bond with the backbone of inulin, while the tails are attached by Leu1 and Lys185. The backbone of the inulin is strongly attracted inside the cavity by the salt bridges. In many other diseases such as COVID-19 IL-6 serve as a target to rescue the host immune response where direct inhibition of these demonstrated the reduced role of IL-6 in viral pathogenesis^62^. Since, our results also block the same residues thus may induce more potent pharmacological properties. The 3D binding mode of inulin the binding cavity of IL-6 is given in Fig. 5a while the 2D detailed interaction pattern of inulin with IL-6 is shown in Fig. 5b.

### Root mean square deviation

**Figure 6:**
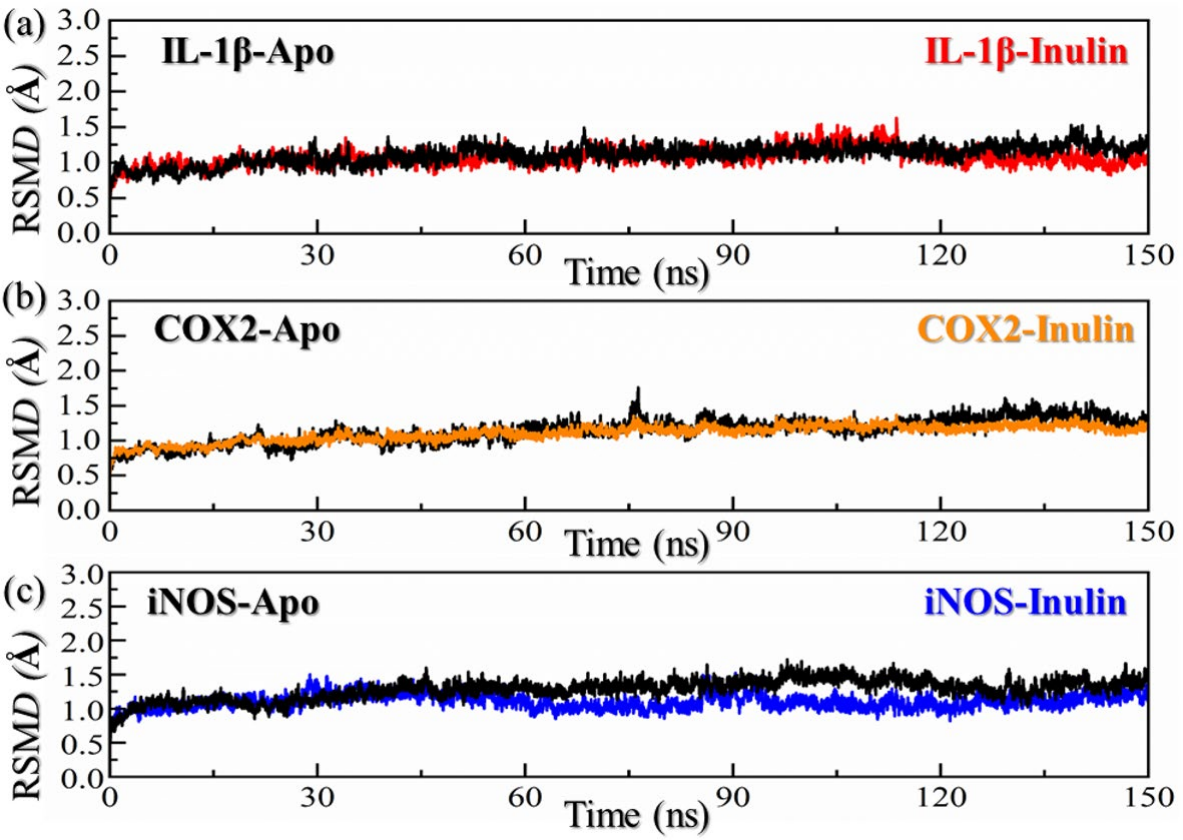
Dynamics-based assessment of stability of IL-1b, COX2 and iNOS in bound states with inulin. (**a**) Show the RMSD results for IL-1b–inulin complex, (**b**) show the RMSD results for COX2–inulin complex while (**c**) represent the RMSD graph for iNOS–inulin complex.

Assessing the dynamic stability of a protein bound to a ligand is essential to demonstrate the pharmacological activity of that particular compound. The compound IL-1β–Inulin stabilized at 1.3 Å. In the case of compound IL-1β–Inulin the RMSD initially remained stable with no major deviation. No significant deviation was observed during the simulation except a minor increment in the RMSD graph at 0–10 and 85–90 ns. Then the RMSD gradually decreased and increased following this pattern until the end of 150 ns simulation. However, the complex demonstrated stable dynamics with an average RMSD of 1.30 Å, because when RMSD ≤ 2 Å can prove the reliability of the docking method. For the COX2–Inulin, the RMSD increased gradually since the beginning. The RMSD had large fluctuations and continues to increase with abrupt increase and decrease between 30 and 60 ns. The COX2–Inulin reported significant structural perturbation after 30 ns, the maximum RMSD value is 4.8 Å. The complex demonstrated a uniform RMSD pattern between 60 and 100 ns with the gradual increasing trend but then significant perturbations were seen in the complex between 100 and 105 ns, the maximum RMSD value is 4.2 Å. Then maintain a steady trend. Because the RMSD fluctuates greatly, the difference between the maximum value and the minimum value is close to 4.0 Å, and the complex demonstrated unstable dynamics with an average RMSD of 3.5 Å. When RMSD ≥ 2 Å can’t prove the reliability of the docking method. In general, the compound IL-1β–Inulin is relatively stable, but COX2–Inulin is unstable. On the other hand, the iNOS–Inulin complex that reported the best docking score among the all with highest number of hydrogen bonds and other interactions also demonstrated stabilized dynamic behavior throughout the simulation. The complex stabilized at 3.0 Å and continues to remain uniform with minor deviation at 75 ns. The complex overall attained a very stable dynamic with an average RMSD of 2.8 Å until the end of simulation. The RMSD for the iNOS, COX-2, and IL-1β Apo forms is shown in Fig. 6. Dynamic stability of the ligand-bound complexes always yields better pharmacological output as witnessed by many studies, and hence our results strongly align with the previous results were stable complexes of the aforementioned results reported stronger inhibition than the less stable^45,46,58,62^. Overall, this shows that inulin could primarily target iNOS and may supplementarily target the COX-2 and IL-1β during the DSS-induced colitis to reduce the role of these inflammatory machines. The RMSD results for the top three complexes are given in Fig. 6a–c.

### Residual flexibility and hydrogen bond analysis

**Figure 7:**
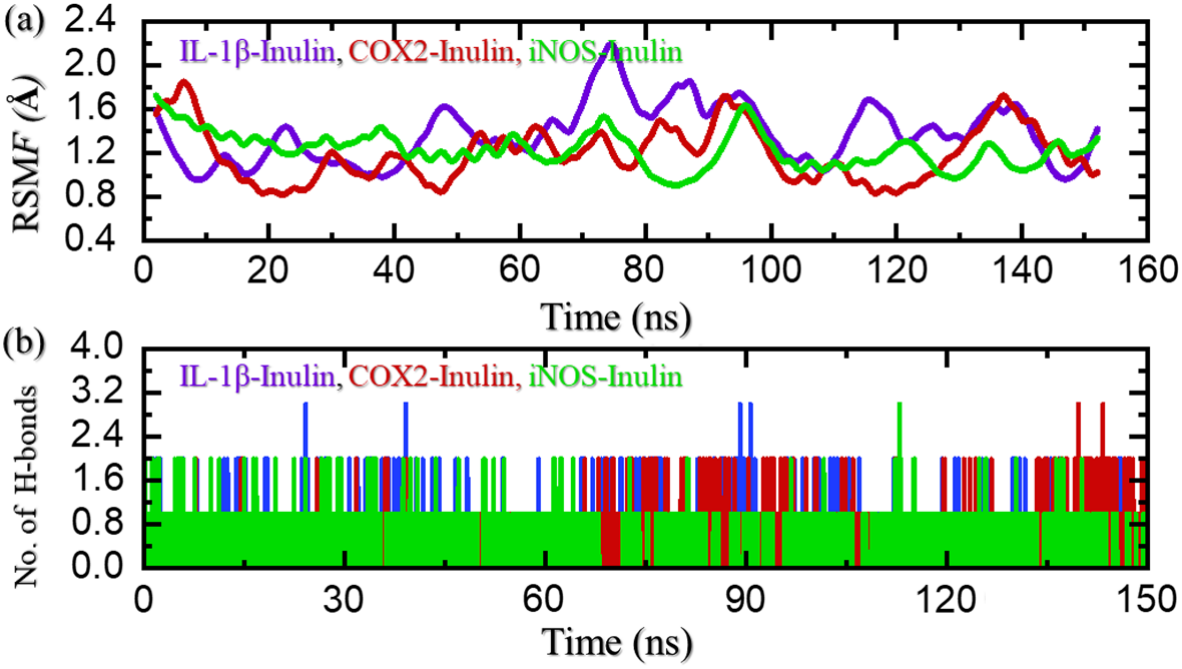
Residue’s flexibility and hydrogen bonding assessment of stability of IL-1β, COX2 and iNOS in bound states with inulin. (**a**) Show the RMSF results for all the complexes, while (**b**) show the H-bonds results for all the complexes.

Root Mean Square Fluctuation (RMSF) analysis was performed to investigate the dynamic behavior of the protein structure throughout the simulation trajectory. The RMSF values were calculated for each residue in the protein. Figure 7a shows the RMSF profile of the protein over specific residues. The x-axis represents the residue number, while the y-axis represents the RMSF values in angstroms. The RMSF values were calculated by measuring the deviation of each residue’s position from its average position during the simulation. The RMSF analysis of IL-1β–Inulin revealed distinct regions of flexibility within the protein structure. Residues 20–25, 42–53, 62–70, and 100–130 exhibited higher RMSF values, indicating increased flexibility compared to other regions. Conversely, residues 0–18, 30–40, and 95–110 showed relatively low RMSF values, suggesting a more rigid conformation. In case of COX2–Inulin, residues 15–30, 40–50, 60–70, and 100–130 exhibited higher RMSF values, indicating increased flexibility compared to other regions. In contrast to IL-1β–Inulin the COX2–Inulin showed lower residual flexibility which may play a crucial role in the protein's function, as increased flexibility in these regions can allow for conformational changes necessary for binding interactions or enzymatic activity. In the case of iNOS–Inulin complex, residues 80–100 demonstrated higher fluctuations while the rest of the regions reported minimal fluctuations.

Hydrogen bonding calculation is one of the key assessments that help in determining the pharmacological potential of a drug/inhibitor. It is an essential approach to reveal the potency and binding strength of the interacting molecules. This approach has been widely applied to understand the pharmacological mechanism of a particular drug, and the interaction mechanism of two or more proteins to reveal the mechanism of a disease or bio-catalytic process^63–67^. Then we performed hydrogen bond analysis to investigate the dynamic behavior and intermolecular interactions within the protein structure throughout the simulation trajectory. Hydrogen bonds were calculated based on distance and angle criteria using a cutoff of 3.5 Å for the donor–acceptor distance and 30° for the donor–hydrogen–acceptor angle. The analysis revealed that all the complexes (IL-1β–Inulin, COX2–Inulin, iNOS–Inulin) maintained several hydrogen bonds within the protein structure in association with the ligand Fig. 7b. Each line represents a hydrogen bond, with the donor and acceptor residues labeled accordingly. Interestingly, in case of IL-1β–Inulin residues involved in hydrogen bonding interactions were found to be consistent with the regions of high flexibility identified through the RMSF analysis. Residues 20–25, 42–53, 62–70, and 100–130, which exhibited higher RMSF values, also showed a higher propensity for forming hydrogen bonds with neighboring residues.

### Radius of gyration

**Figure 8:**
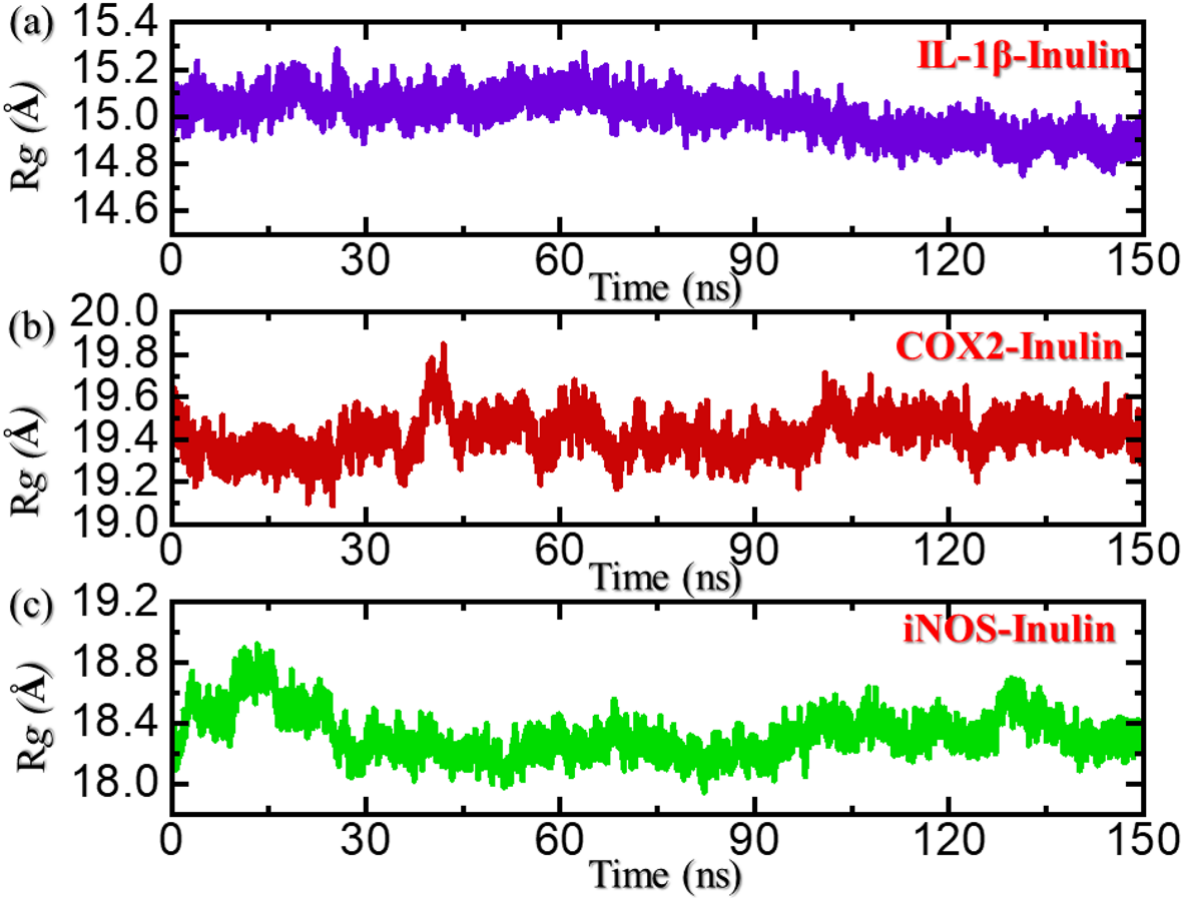
Dynamics-based assessment of structural packing of IL-1b, COX2 and iNOS in bound states with inulin. (**a**) Show the Rg results for IL-1b–inulin complex, (**b**) show the Rg results for COX2–inulin complex while (**c**) represent the Rg graph for iNOS–inulin complex.

Protein packing determination or protein size estimation demonstrates information regarding the essential events that occurred during the molecular simulation. We estimated the structural compactness of each complex during simulation by calculating the radius of gyration (Rg) as a function of time. At the beginning, the Rg of the compound IL-1β–Inulin remained stable, the Rg continued to increase with abrupt increase and decrease between 20 and 30 ns, and there was a maximum value between 25 and 30 ns, with a maximum value of 15.3 Å. The Rg remained stable with no major deviation between 30 and 65 ns. Starting from 65 ns, the Rg pattern also reported a gradual decrease in the Rg trajectory. The Rg started from 15.00 Å, and reached to a maximum of 14.80 Å at 150 ns. An average Rg was calculated to 15.00 Å, thus convey a stable binding of the ligand with no significantly increase or decrease in the protein size during simulation. And the Rg image of the COX2–Inulin is not very stable, showing significant fluctuations overall. The Rg for the COX2–Inulin complex started to decrease initially and reached 19.20 Å at 5 ns and then equilibrated. A straight uniform Rg was seen until 35 ns and then an abrupt increase was observed in the Rg pattern, and reached to a maximum of 19.8 Å. Subsequent Rg also fluctuated at 60 ns, 100 ns, and 125 ns. This shows significant structural perturbation occurred during the simulation and thus causes binding and unbinding of the ligand in the pocket, and an average Rg was calculated to 19.30 Å. Moreover, the Rg for iNOS–Inulin started to increase initially and reached to 18.8 Å, however they started to decrease gradually and attained the tighter structural packing at 30 ns. The Rg continues to follow the same pattern until 130 ns but a small perturbation was observed for a shorter period and then again decreased back. An average Rg for the iNOS–Inulin complex was calculated to be 18.45 Å. The Rg results for the top complexes are given in Fig. 8a–c.

### Clustering of protein’s motion

**Figure 9:**
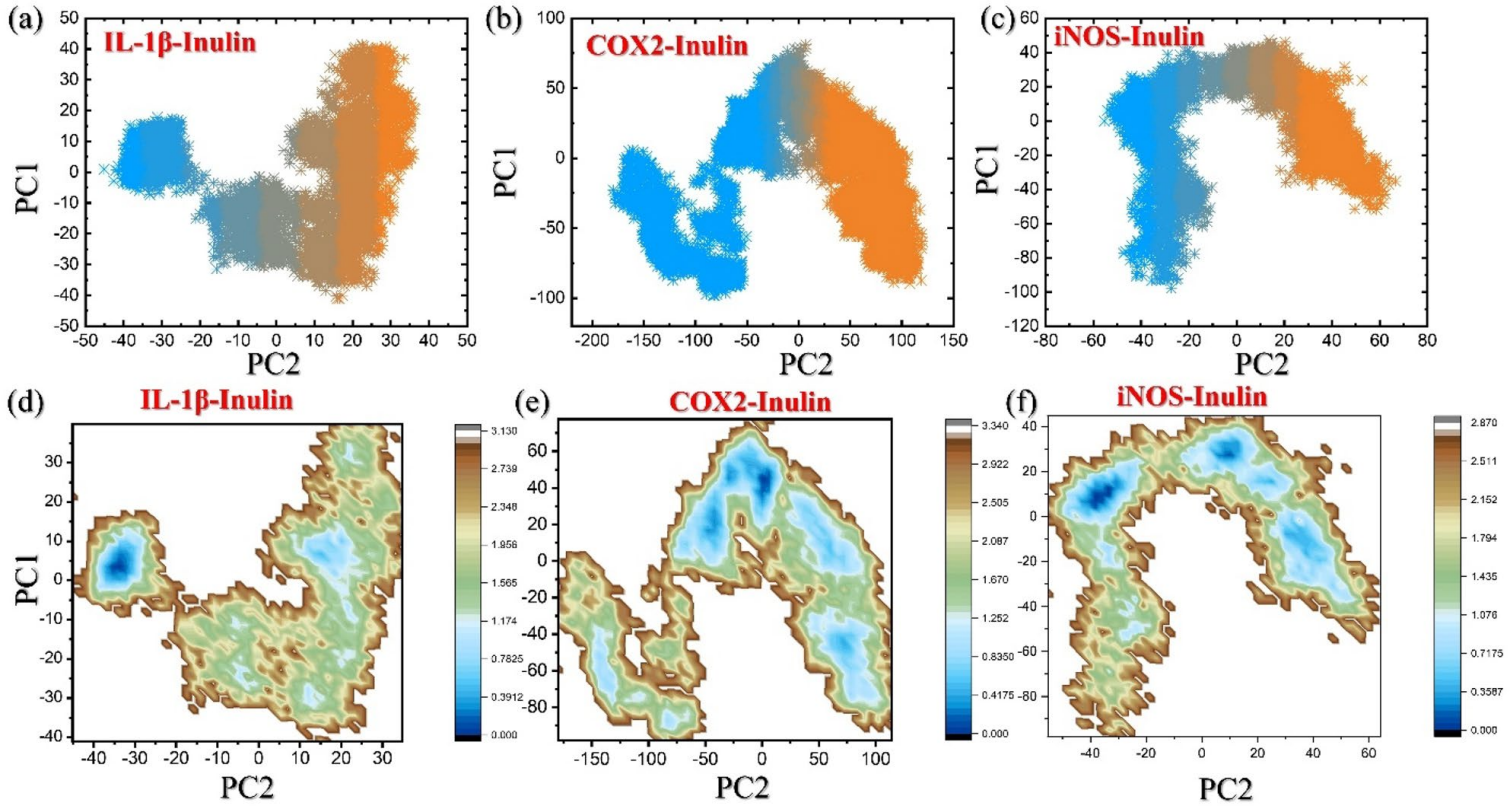
Dynamics-based assessment of trajectories clustering and Free energy landscape of IL-1b, COX2 and iNOS in bound states with inulin. (**a**–**c**) Show the PCA results for IL-1β, COX2 and iNOS in bound states with inulin complexes, while (**b**) show the FEL results for IL-1β, COX2 and iNOS in bound states with inulin complexes.

The protein motions within the simulation trajectories were clustered utilizing principal component analysis (PCA). Each system structural modifications brought about by mutation were plotted using principal component analysis (PCA). Utilizing a trajectory of 0–150 ns MD simulation, PCA was performed to learn about the conformational states of the IL-1β–Inulin, COX2–Inulin and iNOS–Inulin complexes. The eigenvalues of the covariance matrix served as the foundation for conducting principal component analysis (PCA), enabling the assessment of the overall motion of Cα-atoms in the hits and complexes. The motions observed in two dimensions were represented by PC1 and PC2. Figure 9a–c illustrates the distributed principal components for each complex. The dots represent the various frames, which progress from orange to indigo. The IL-1β–Inulin approximately 70.0% of the total motion was attributed to the first three eigenvectors, while the remaining motion was contributed by the remaining eigenvectors. In contrast, for the COX2–Inulin eigenvectors contributed 75.0% to the total motion. Furthermore, the iNOS–Inulin contributed 60.0% of the total motion by the first three eigenvectors. The conformational transition between them can be clearly distinguished by the orange and indigo colors. As a result, the overall internal motion is reduced, leading to effective binding effects.

In order to explore thermodynamically favorable changes upon the mutation, motion mode analysis was carried out. PCA was applied using the covariance matrix of the 3D positional coordinates of different IL-1β–Inulin and COX2–Inulin complexes obtained from a 150 ns MD simulation. This allowed identification of the principal components (PCs) representing specific motion modes performed by these complex systems. The first PC captured the dominant motion observed in the analysis. Figure 9d–f demonstrate that the IL-1β–Inulin achieved a single conformation with the lowest energy, COX2–Inulin and iNOS–Inulin exhibited two lowest-energy conformations.

### MM/GBSA analysis

Molecular Mechanics/Generalized Born Surface Area (MM/GBSA) analysis was performed to assess the binding free energy and contribute to our understanding of the protein–ligand interactions. The MM/GBSA calculations were carried out for the protein–ligand complexes sampled during the molecular dynamic’s simulations. Table 1 summarizes the calculated MM/GBSA energy components for each protein–ligand complex. The binding free energy (ΔG_bind) is represented as the sum of the contributions from different terms, including the molecular mechanics energy (ΔG_MM), the solvation energy (ΔG_GBSA), and the entropy contribution (ΔS). The standard deviation (SD) of the calculated values is also provided to indicate the reliability of the results. The calculated ΔG_bind values indicate the overall binding affinity of the ligands to the protein. Negative ΔG_bind values suggest favorable binding, while positive values indicate unfavorable binding. In our study, IL-1β–Inulin and COX2–Inulin displayed ΔG bind values of − 27.76 kcal/mol and − 37.78 kcal/mol, while the iNOS–Inulin demonstrated the best binding free energy results, − 45.89 kcal/mol, in contrast to the first two complex, respectively, indicating strong binding affinities. Further, their individual energy contribution was calculated which shows the residues Trp194, Arg199, Ile201, Gly202, Ser242, Phe369, Asn370, Gly371 and Trp372. The total energy contribution of these residues showed correlation of − 011, − 0.016, − 0.17, − 0.047, − 0.85, − 0.072, − 0.48, − 0.44, and − 0.034 respectively. Overall, the MM/GBSA analysis provided valuable insights into the binding free energy and the key molecular interactions between the protein and ligand. The results provide significant insights into the inhibitory potential of these targets by BFO and are showing better results than others^7,58,68^. These findings enhance our understanding of the structure–activity relationships and guide future drug design efforts targeting the protein of interest.

**Table 1:** Binding free energy results for the three complexes estimated using MM/GBSA approach and reported in kcal/mol.

Parameters	IL-1β–Inulin	COX2–Inulin	iNOS–Inulin
ΔEvdw	− 44.09 ± 3.21	− 54.05 ± 5.95	− 68.19 ± 0.20
ΔEele	− 17.00 ± 6.44	− 7.70 ± 2.71	− 6.36 ± 0.13
ESURF	− 5.77 ± 0.38	− 7.34 ± 0.64	37.13 ± 0.17
EGB	39.11 ± 6.05	31.32 ± 4.09	− 8.48 ± 0.02
∆G total	− 27.76 ± 2.97	− 37.78 ± 4.52	− 45.89 ± 0.16

## Discussion

Pro-inflammatory cytokines exert a central regulatory role in the pathogenesis and advancement of DSS-induced colitis^69^. In our experimental study, we observed a significant increase in the mRNA expression levels of pro-inflammatory cytokines, including IL-1β, IL-6, and TNF-α, following DSS administration, indicative of colitis induction in mice. However, intriguingly, administration of inulin led to a remarkable reduction in the upregulation of these cytokines. This suggests that inulin exerts a suppressive effect on the inflammatory response associated with DSS-induced colitis.

Similarly, the expression of inducible nitric oxide synthase (iNOS) and cyclooxygenase-2 (Cox-2) markedly increased following administration of dextran sulfate sodium (DSS). However, varied concentrations of inulin exhibited a dose-dependent reduction in the expression of these proinflammatory cytokines. This suggests a significant mitigating effect of inulin on the inflammatory response induced by DSS treatment^25^. iNOS and COX‐2 play crucial roles in inflammatory signaling pathways, triggering the synthesis of nitric oxide (NO) and prostaglandin E2 (PGE2) in the mucosa. This activation can contribute to an increase in inflammatory cytokines such as TNF‐α, IL‐1β, and IL‐6, thereby intensifying inflammation and causing tissue damage^70,71^.

In a previous study, buddlejasaponin IV demonstrated the capacity to diminish the expression levels of proinflammatory proteins such as iNOS and COX-2, alongside the mRNA expression of cytokines TNF-α, IL-1β, and IL-6 in RAW264.7 cells. This effect was achieved through the inhibition of the NF-κB signaling pathway, elucidating its potential as a regulator of inflammation at the molecular level. In cellular processes, the transcription factor NF-κB is intricately regulated by its association with an inhibitory subunit, IκB, predominantly located in the cytoplasm in an inert state. Upon stimulation, IκB undergoes phosphorylation, triggering its degradation and facilitating the translocation of NF-κB into the nucleus. Once in the nucleus, NF-κB orchestrates the transcriptional activation of genes responsible for various cellular functions. Notably, the compound buddlejasaponin IV exhibited a concentration-dependent inhibition of NF-κB translocation, thereby modulating its activity in the nucleus^72^. In our investigation we examined the possibility that inulin inhibits NF-κb activity in a similar pattern.

Liu et al. conducted an investigation into the synergistic impact of inulin in combination with *Lactobacillus* *rhamnosus* using a murine model of DSS-induced colitis. Their study revealed the potent anti-inflammatory attributes of inulin. Elevated levels of proinflammatory cytokines, including IL-1β, IL-6, and TNF-α, were observed in the DSS-treated group, a response mitigated upon the administration of inulin combined with *Lactobacillus* *rhamnosus*. Moreover, this combination led to an enhancement in the abundance and diversity of intestinal microbiota. Notably, alterations in the microbiota composition may indirectly influence iNOS levels, indicating a potential mechanism underlying the observed anti-inflammatory effects. Inulin is fermented by gut bacteria in the colon, generating short-chain fatty acids (SCFAs), including metabolites. SCFAs, particularly, serve as energy sources for intestinal cells and regulate immune cell differentiation^73,74^. Additionally, in stress-recurrent inflammatory bowel disease (IBD), inulin was found to improve symptoms by modulating microbiota composition and reducing inflammation, which may involve the regulation of iNOS. Inulin decreases the serum inflammatory markers IL-6, ACALP and inflammatory cytokines IL6 in colon and express immunomodulatory effect^75^ Inulin is a probiotic that alter the microbiota of the intestine and suppress the inflammation by inhibiting the expression of tumor necrosis factor related cytokines^76^. We employed a multifaceted computational approach to elucidate potential molecular targets for Burdock Inulin, particularly focusing on inflammatory proteins implicated in conditions like DSS-induced colitis. Our findings from molecular docking and simulation shed light on the interaction dynamics between Inulin and key inflammatory proteins including iNOS, COX-2, TNF-alpha, IL-6, and IL-1β. The molecular docking results revealed favorable interactions and binding affinities between Inulin and the selected targets. Notably, IL-1β, COX-2, and iNOS emerged as the most promising targets based on their binding scores and interaction profiles. These findings corroborate previous studies suggesting the potential anti-inflammatory properties of Inulin.

## Conclusions

Target validation using in silico computational modeling approaches was employed and validated through molecular simulation-based methods. Our results identified IL-1β, COX2, and iNOS as the best biotargets for inulin. However, simulation-based further validations revealed that among all, iNOS serves as the best target for inulin. The interaction of inulin with iNOS demonstrated stable dynamics, a hydrogen bonding paradigm, and dynamic motion properties. Thus, further experiments are needed to use inulin to target iNOS and reduce DSS-induced colitis and other autoimmune diseases.

## Data Availability

Data will be available upon request from the corresponding author.

## References

[1] McComb S (2019). Introduction to the immune system. Immunoproteomics Methods Protoc..

[2] Zmora N (2017). The role of the immune system in metabolic health and disease. Cell Metab..

[3] Liu C (2021). Cytokines: From clinical significance to quantification. Adv. Sci..

[4] Tayal V, Kalra BS (2008). Cytokines and anti-cytokines as therapeutics—An update. Eur. J. Pharmacol..

[5] Dinarello CA (2000). Proinflammatory cytokines. Chest.

[6] Ramani T (2015). Cytokines: The good, the bad, and the deadly. Int. J. Toxicol..

[7] Conti P (2020). Induction of pro-inflammatory cytokines (IL-1 and IL-6) and lung inflammation by Coronavirus-19 (COVI-19 or SARS-CoV-2): Anti-inflammatory strategies. J. Biol. Regul. Homeost. Agents.

[8] Idriss HT, Naismith JH (2000). TNFα and the TNF receptor superfamily: Structure-function relationship (s). Microsc. Res. Tech..

[9] Opal SM, DePalo VA (2000). Anti-inflammatory cytokines. Chest.

[10] Balkwill F (2006). TNF-α in promotion and progression of cancer. Cancer Metastasis Rev..

[11] Moelants EA (2013). Regulation of TNF-α with a focus on rheumatoid arthritis. Immunol. Cell Biol..

[12] Brietzke E, Kapczinski F (2008). TNF-α as a molecular target in bipolar disorder. Prog. Neuro-Psychopharmacol. Biol. Psychiatry.

[13] Wang R (2018). iNOS promotes CD24+ CD133+ liver cancer stem cell phenotype through a TACE/ADAM17-dependent Notch signaling pathway. Proc. Natl. Acad. Sci..

[14] Kielbik M, Szulc-Kielbik I, Klink M (2019). The potential role of iNOS in ovarian cancer progression and chemoresistance. Int. J. Mol. Sci..

[15] Rocca B (2002). Cyclooxygenase-2 expression is induced during human megakaryopoiesis and characterizes newly formed platelets. Proc. Natl. Acad. Sci..

[16] Takemiya T (2003). Inducible brain COX-2 facilitates the recurrence of hippocampal seizures in mouse rapid kindling. Prostaglandins Other Lipid Mediat..

[17] Lopez-Castejon G, Brough D (2011). Understanding the mechanism of IL-1β secretion. Cytokine Growth Factor Rev..

[18] Ribeiro VP (2018). Brazilian medicinal plants with corroborated anti-inflammatory activities: A review. Pharm. Biol..

[19] Singh S (2020). Medicinal plants used against various inflammatory biomarkers for the management of rheumatoid arthritis. J. Pharm. Pharmacol..

[20] Chan Y-S (2011). A review of the pharmacological effects of *Arctium*
*lappa* (burdock). Inflammopharmacology.

[21] Duh PD (1998). Antioxidant activity of burdock (*Arctium*
*lappa* Linne): Its scavenging effect on free-radical and active oxygen. J. Am. Oil Chem. Soc..

[22] Chen F-A, Wu A-B, Chen C-Y (2004). The influence of different treatments on the free radical scavenging activity of burdock and variations of its active components. Food Chem..

[23] Lou Z (2010). Assessment of antibacterial activity of fractions from burdock leaf against food-related bacteria. Food Control.

[24] Zhang X-J (2019). Immunomodulatory activity of a fructooligosaccharide isolated from burdock roots. RSC Adv..

[25] Ma Q (2023). Long-term oral administration of burdock fructooligosaccharide alleviates DSS-induced colitis in mice by mediating anti-inflammatory effects and protection of intestinal barrier function. Immun. Inflamm. Dis..

[26] Scholz C (2015). DOCKTITE—A highly versatile step-by-step workflow for covalent docking and virtual screening in the molecular operating environment. J. Chem. Inf. Model..

[27] Guex N, Peitsch MC (1997). SWISS-MODEL and the Swiss-Pdb Viewer: An environment for comparative protein modeling. Electrophoresis.

[28] Attique SA (2019). A molecular docking approach to evaluate the pharmacological properties of natural and synthetic treatment candidates for use against hypertension. Int. J. Environ. Res. Public Health.

[29] Clark AM, Labute P (2007). 2D depiction of protein–ligand complexes. J. Chem. Inf. Model..

[30] Kumari R, Dalal V (2022). Identification of potential inhibitors for LLM of *Staphylococcus*
*aureus*: Structure-based pharmacophore modeling, molecular dynamics, and binding free energy studies. J. Biomol. Struct. Dyn..

[31] Kumari R (2022). Structural-based virtual screening and identification of novel potent antimicrobial compounds against YsxC of *Staphylococcus*
*aureus*. J. Mol. Struct..

[32] Land H, Humble MS (2018). YASARA: A tool to obtain structural guidance in biocatalytic investigations. Protein Engineering.

[33] Wang J (2004). Development and testing of a general amber force field. J. Comput. Chem..

[34] Yin L-L (2020). Molecular dynamics simulation and kinetic study of fluoride binding to V21C/V66C myoglobin with a cytoglobin-like disulfide bond. Int. J. Mol. Sci..

[35] Chen S (2021). Molecular dynamic simulations of bromodomain and extra-terminal protein 4 bonded to potent inhibitors. Molecules.

[36] Xu W (2020). All-atomic molecular dynamic studies of Human and Drosophila CDK8: Insights into their kinase domains, the LXXLL motifs, and drug binding site. Int. J. Mol. Sci..

[37] Roe DR, Cheatham TE (2013). PTRAJ and CPPTRAJ: Software for processing and analysis of molecular dynamics trajectory data. J. Chem. Theory Comput.

[38] Chen F (2016). Assessing the performance of the MM/PBSA and MM/GBSA methods. 6. Capability to predict protein–protein binding free energies and re-rank binding poses generated by protein–protein docking. Phys. Chem. Chem. Phys..

[39] Dalal V (2021). Structure-based identification of potential drugs against FmtA of *Staphylococcus*
*aureus*: Virtual screening, molecular dynamics, MM-GBSA, and QM/MM. Protein J..

[40] Teli MK (2021). In silico identification of hydantoin derivatives: A novel natural prolyl hydroxylase inhibitor. J. Biomol. Struct. Dyn..

[41] Li J (2011). The VSGB 2.0 model: A next generation energy model for high resolution protein structure modeling. Proteins.

[42] Khan A (2020). Dynamics insights into the gain of flexibility by helix-12 in ESR1 as a mechanism of resistance to drugs in breast cancer cell lines. Front. Mol. Biosci..

[43] Khan A (2021). Higher infectivity of the SARS-CoV-2 new variants is associated with K417N/T, E484K, and N501Y mutants: An insight from structural data. J. Cell. Physiol..

[44] Khan A (2022). The Omicron (B.1.1.529) variant of SARS-CoV-2 binds to the hACE2 receptor more strongly and escapes the antibody response: Insights from structural and simulation data. Int. J. Biol. Macromol..

[45] Khan A (2019). Deep-learning-based target screening and similarity search for the predicted inhibitors of the pathways in Parkinson's disease. RSC Adv..

[46] Khan A (2023). Discovery of Isojacareubin as a covalent inhibitor of SARS-CoV-2 main protease using structural and experimental approaches. J. Med. Virol..

[47] Khan A (2021). In silico and in vitro evaluation of kaempferol as a potential inhibitor of the SARS-CoV-2 main protease (3CLpro). Phytother. Res..

[48] Khan A (2022). The Omicron (B.1.1.529) variant of SARS-CoV-2 binds to the hACE2 receptor more strongly and escapes the antibody response: Insights from structural and simulation data. Int. J. Biol. Macromol..

[49] Khan A (2023). Structure-based design of promising natural products to inhibit thymidylate kinase from Monkeypox virus and validation using free energy calculations. Comput. Biol. Med..

[50] Nair MS, Shukla A (2020). Molecular modeling, simulation and principal component analysis of binding of resveratrol and its analogues with DNA. J. Biomol. Struct. Dyn..

[51] Bao H (2023). Probing mutation-induced conformational transformation of the GTP/M-RAS complex through Gaussian accelerated molecular dynamics simulations. J. Enzyme Inhib. Med. Chem..

[52] Dash R (2019). Structural and dynamic characterizations highlight the deleterious role of SULT1A1 R213H polymorphism in substrate binding. Int. J. Mol. Sci..

[53] Chen J (2021). Mutation-induced impacts on the switch transformations of the GDP-and GTP-bound K-ras: Insights from multiple replica Gaussian accelerated molecular dynamics and free energy analysis. J. Chem. Inf. Model..

[54] Lee S-B (2017). Xanthotoxin suppresses LPS-induced expression of iNOS, COX-2, TNF-α, and IL-6 via AP-1, NF-κB, and JAK-STAT inactivation in RAW 264.7 macrophages. Int. Immunopharmacol..

[55] Sakthivel KM, Guruvayoorappan C (2013). *Acacia*
*ferruginea* inhibits tumor progression by regulating inflammatory mediators-(TNF-α, iNOS, COX-2, IL-1β, IL-6, IFN-γ, IL-2, GM-CSF) and pro-angiogenic growth factor-VEGF. Asian Pac. J. Cancer Prev..

[56] Guo Y (2018). Dihydrotanshinone I, a natural product, ameliorates DSS-induced experimental ulcerative colitis in mice. Toxicol. Appl. Pharmacol..

[57] Feng Y (2022). Pectolinarigenin suppresses LPS-induced inflammatory response in macrophages and attenuates DSS-induced colitis by modulating the NF-κB/Nrf2 signaling pathway. Inflammation.

[58] Fang Y (2023). Network pharmacology-and molecular simulation-based exploration of therapeutic targets and mechanisms of heparin for the treatment of sepsis/COVID-19. J. Biomol. Struct. Dyn..

[59] Ahmad A (2018). Ursolic acid rich *Ocimum*
*sanctum* L. leaf extract loaded nanostructured lipid carriers ameliorate adjuvant induced arthritis in rats by inhibition of COX-1, COX-2, TNF-α and IL-1: Pharmacological and docking studies. PLoS One.

[60] Kerdphon S (2023). Structure-activity relationship and molecular docking of quinazolinones inhibiting expression of COX-2, IL-1β, iNOS, and TNF-α through NF-κB pathways. ACS Med. Chem. Lett..

[61] Razak S (2021). Molecular docking, pharmacokinetic studies, and in vivo pharmacological study of indole derivative 2-(5-methoxy-2-methyl-1H-indole-3-yl)-N′-[(E)-(3-nitrophenyl) methylidene] acetohydrazide as a promising chemoprotective agent against cisplatin induced organ damage. Sci. Rep..

[62] Nada H (2023). Perspective for discovery of small molecule IL-6 inhibitors through study of structure-activity relationships and molecular docking. J. Med. Chem..

[63] Khan A (2022). Structural and molecular insights into the mechanism of resistance to enzalutamide by the clinical mutants in androgen receptor (AR) in castration-resistant prostate cancer (CRPC) patients. Int. J. Biol. Macromol..

[64] Prekovic S (2016). The effect of F877L and T878A mutations on androgen receptor response to enzalutamide molecular analysis of androgen receptor mutants. Mol. Cancer Ther..

[65] Selvaraj D (2021). Syringaresinol as a novel androgen receptor antagonist against wild and mutant androgen receptors for the treatment of castration-resistant prostate cancer: Molecular docking, in-vitro and molecular dynamics study. J. Biomol. Struct. Dyn..

[66] Hu X (2020). Advances in the computational development of androgen receptor antagonists. Drug Discov. Today.

[67] Gim HJ (2021). Conformational dynamics of androgen receptors bound to agonists and antagonists. Sci. Rep..

[68] Dalal V, Golemi-Kotra D, Kumar P (2022). Quantum mechanics/molecular mechanics studies on the catalytic mechanism of a novel esterase (FmtA) of *Staphylococcus*
*aureus*. J. Chem. Inf. Model..

[69] Andújar I (2011). Inhibition of ulcerative colitis in mice after oral administration of a polyphenol-enriched cocoa extract is mediated by the inhibition of STAT1 and STAT3 phosphorylation in colon cells. J. Agric. Food Chem..

[70] Soufli I (2023). Nitric oxide, neutrophil/lymphocyte, and platelet/lymphocyte ratios as promising inflammatory biomarkers in complicated Crohn’s disease: Outcomes of corticosteroids and anti-TNF-α therapies. Inflammation.

[71] Wang M (2023). Dietary lactate supplementation can alleviate DSS-induced colitis in piglets. Biomed. Pharmacother..

[72] Won J-H (2006). Anti-inflammatory effect of buddlejasaponin IV through the inhibition of iNOS and COX-2 expression in RAW 264.7 macrophages via the NF-κB inactivation. Br. J. Pharmacol..

[73] Liu Z (2020). Study of the alleviation effects of a combination of *Lactobacillus*
*rhamnosus* and inulin on mice with colitis. Food Funct..

[74] Sheng W, Ji G, Zhang L (2023). Immunomodulatory effects of inulin and its intestinal metabolites. Front. Immunol..

[75] Du Y (2024). Protective effects of inulin on stress-recurrent inflammatory bowel disease. Int. J. Mol. Sci..

[76] Roy S, Dhaneshwar S (2023). Role of prebiotics, probiotics, and synbiotics in management of inflammatory bowel disease: Current perspectives. World J. Gastroenterol..

